# Risk factors of mortality in severe cutaneous adverse reactions patients with pulmonary involvement

**DOI:** 10.1186/cc10726

**Published:** 2012-03-20

**Authors:** H Hu, K Kao, C Huang, C Yang, C Hung

**Affiliations:** 1 Chang Gung Memorial Hospital, Taoyuan, Taiwan

## Introduction

Severe cutaneous adverse reactions, such as Stevens-Johnson syndrome/toxic epidermal necrolysis (SJS/TEN) and drug reaction with eosinophilia and systemic symptoms (DRESS) are uncommon but potentially critical ills. Pulmonary involvements in these severe cutaneous reactions patients are rare but are life-threatening complications. Therefore, we conducted a study to investigate the outcomes and risk factors of patients with severe cutaneous reactions with pulmonary complications.

## Methods

This is a retrospective study conducted in a tertiary teaching hospital in Taiwan. Between September 2002 and June 2011, 23 consecutive patients admitted to our hospital under the diagnosis of severe cutaneous adverse reactions with pulmonary involvements were enrolled. The collected demographic data included gender, age and comorbidity. Laboratory data and possible offending etiology also were collected by reviewing the medical records.

## Results

A total of 21 severe cutaneous adverse reactions patients were eligible. In these 21 patients, 16 (76.2%) patients were SJS/TEN and five (23.8%) patients were DRESS. Allopurinol was the most common culprit medicine (*n *= 9). There were 11 (52.4%) patients progressing to respiratory failure with mechanical ventilation. Among these 11 patients, one was upper airway obstruction, two patients were pneumonia, three patients were acute respiratory distress syndrome and the other five patients were acute pulmonary edema. The overall hospital mortality rate was 47.6% (11/21). The survivors group was younger (51.5 ± 25.4 years vs. 70 ± 10.7 years, *P *= 0.046) and had less chronic kidney disease (9% vs. 60%, *P *= 0.021) compared with the nonsurvivors group.

## Conclusion

Severe cutaneous adverse reaction with lung involvement may contribute a high mortality rate. Older age and comorbidity of chronic kidney disease were the risk factors of mortality in severe cutaneous adverse reactions patients.

